# Redefining Idiopathic Normal Pressure Hydrocephalus Using AI-Driven Brain Volumetry

**DOI:** 10.3390/biomedicines14030677

**Published:** 2026-03-16

**Authors:** Juan Sahuquillo, Murad Al-Nusaif, Aasma Sahuquillo-Muxi, Paula Duch, Maria-Antonia Poca, on behalf of the Alzheimer’s Disease Neuroimaging Initiative

**Affiliations:** 1 Department of Neurosurgery, Vall d’Hebron University Hospital (VHUH), Vall d’Hebron Barcelona Hospital Campus, Passeig Vall d’Hebron 119-129, 08035 Barcelona, Spain; pocama@neurotrauma.net; 2 Neurotraumatology and Neurosurgery Research Unit, Vall d’Hebron Institut de Recerca (VHIR), c, Passeig Vall d’Hebron 119-129, 08035 Barcelona, Spain; murad.alnusaif@icfo.eu (M.A.-N.); aasmasm@outlook.com (A.S.-M.); pauladuchvega@gmail.com (P.D.); 3 Department of Surgery, Vall d’Hebron Barcelona Hospital Campus, Universitat Autònoma de Barcelona, 08035 Barcelona, Spain; 4 ICFO-Institut de Ciències Fotòniques, 08860 Castelldefels, Spain

**Keywords:** idiopathic normal pressure hydrocephalus, MRI volumetry, artificial intelligence, ventriculomegaly, neurodegeneration, brain atrophy

## Abstract

Idiopathic normal pressure hydrocephalus (iNPH) is a potentially reversible cause of gait disturbance and cognitive impairment in older adults, yet its diagnosis remains challenging and controversial. The core difficulty lies in distinguishing true hydrocephalus from ventricular enlargement secondary to cerebral atrophy or neurodegenerative disease, a distinction now recognized as non-binary. In many patients, ventricular enlargement reflects a continuum ranging from predominantly hydrocephalic iNPH to mixed pathological states combining impaired cerebrospinal fluid (CSF) dynamics and neurodegeneration. Conventional neuroradiological markers, including the Evans Index, the callosal angle, and the disproportionately enlarged subarachnoid-space hydrocephalus (DESH) pattern, provide useful qualitative guidance but are limited by their two-dimensional nature, interobserver variability, and poor sensitivity for differential diagnosis and outcome prediction. Over the past decade, advances in artificial intelligence-based brain volumetry (AI-BrV) have introduced a new paradigm for quantitative structural assessment. By enabling automated, anatomically precise, and reproducible three-dimensional quantification of ventricular and extraventricular CSF, cortical and subcortical gray matter, deep gray matter nuclei, and periventricular white matter, AI-BrV addresses many limitations of traditional imaging approaches. Beyond absolute volume measurements, AI-BrV enables the derivation of composite indices and ratios that may capture disease-specific structural phenotypes and better reflect the underlying pathophysiology of ventricular enlargement. Importantly, AI-BrV pipelines can be applied retrospectively to large legacy neuroimaging datasets and compared with extensive publicly available repositories, facilitating normative modeling, cross-disease analyses, and external validation of volumetric biomarkers. When integrated with clinical data and multivariable statistical or machine-learning frameworks, these approaches hold promise for improving patient selection, refining disease categorization, and supporting more rational decision-making regarding CSF diversion. In this context, AI-BrV offers a unifying framework for reconciling divergent clinical perspectives and advancing iNPH toward a more precise, reproducible, and evidence-based diagnostic and therapeutic paradigm.

## 1. Introduction

According to the Global Burden of Disease Study 2021 (GBD 2021), global life expectancy increased by 22.7 years between 1950 and 2021 [1]. This substantial gain in longevity—particularly in middle- and high-income countries—has been driven largely by higher educational attainment, rising per capita income, and sustained investments in health promotion and disease prevention [2]. Declining fertility rates, coupled with increasing life expectancy, are profoundly reshaping global population age structures, driving a substantial worldwide increase in the prevalence of cognitive impairment and dementia [2]. Age-specific prevalence models indicate that dementia affects approximately 7–8% of individuals aged 65 years or older, with the highest prevalence observed in high-income countries, largely attributable to longer life expectancy and more comprehensive diagnostic coverage [2,3]. Globally, the number of people living with dementia is expected to triple by 2050 [1]. These projections underscore the critical importance of identifying potentially reversible causes of cognitive decline, among which idiopathic normal pressure hydrocephalus (iNPH) represents a particularly relevant and treatable condition.

First described by Hakim and Adams in 1965 [4], iNPH is a form of communicating hydrocephalus and a frequently underrecognized cause of gait disturbance, cognitive impairment, and urinary incontinence in older adults, which may be partially—or, in selected cases, substantially—reversible with appropriate surgical treatment. For many years, evidence has supported favorable outcomes in selected patients with “probable” iNPH—a diagnostic category widely used in clinical guidelines, alongside “possible” and “confirmed” iNPH [5]. Evidence from retrospective studies, prospective non-randomized cohorts, and a limited number of randomized clinical trials consistently indicates that shunt surgery confers clinical benefit to the majority of appropriately selected patients [6,7,8]. However, despite a growing body of evidence, the validity of idiopathic normal pressure hydrocephalus as a distinct clinical entity has been questioned by several members of the neurology community [9,10,11,12].

Espay et al. highlighted this ongoing skepticism by critiquing the current diagnostic standard for iNPH. They noted that, “*Despite the many pitfalls associated with a diagnosis whose gold standard is, paradoxically, the short-term response to cerebrospinal fluid (CSF) drainage, there remains a misconception that NPH is among the most common treatable causes of dementia in the elderly.*” [9]. This position is particularly striking in light of recent randomized clinical trials and the Cochrane systematic review by Pearce et al., which demonstrated that patients over 60 years of age presenting with gait disturbance, cognitive impairment, and urinary incontinence, together with radiological evidence of ventriculomegaly, experience clear benefit from shunt surgery [7,13]. As Fasano remarked in a recent editorial, iNPH is frequently regarded by neurologists as a “neurosurgical disorder,” with the neurology and neurosurgical communities often divided between believers and agnostics [14]. Most clinicians within the “believers’ camp”—predominantly neurosurgeons—agree that patients may benefit from CSF shunting if they meet specific diagnostic criteria. These criteria typically include the presence of at least one symptom of the classical triad—gait abnormalities, cognitive impairment, and urinary incontinence—and evidence of ventricular dilatation, often supported by neuroimaging markers such as an Evans Index (EI) > 0.30, a reduced callosal angle, or the disproportionately enlarged subarachnoid-space hydrocephalus (DESH) pattern [5]. By contrast, skeptics contend that in many patients ventricular enlargement reflects underlying neurodegenerative pathology and represents an epiphenomenon of disease—*ex vacuo* ventriculomegaly secondary to progressive brain atrophy—rather than a primary, treatable disturbance of CSF dynamics. This perspective—albeit in a more optimistic formulation—was also reflected in the work of several neurosurgeons in the 1960s who, shortly after the seminal publication by Hakim et al., suggested that selected cases of *ex vacuo* ventricular dilatation associated with parenchymal loss could nonetheless exhibit meaningful clinical improvement following CSF shunting [15].

For those of us in the so-called “believers’ camp,” years of accumulated clinical and research experience—including detailed neuropsychological assessment, diverse neuroimaging markers, intracranial pressure (ICP) monitoring, CSF dynamic studies, and the use of a wide range of shunt systems—have underscored several fundamental questions that remain unresolved [16]. These challenges include the marked heterogeneity of ICP profiles in iNPH, the limited reliability of current screening criteria for early case identification, the absence of standardized diagnostic frameworks for establishing probable iNPH, and the critical need for neurosurgeons to make informed, evidence-based decisions when selecting CSF shunt systems—decisions that directly influence surgical morbidity and long-term neurological outcomes [8,16,17]. In addition, clinical outcomes following surgery for iNPH are heterogeneous: improvement may be absent, modest, or transient in some patients and a subset ultimately deteriorates over the long term despite an apparently functioning shunt [9,11]. These observations raise several fundamental questions that remain unresolved, particularly with respect to disease definition, pathophysiology, and patient stratification.

## 2. Differentiating *Ex Vacuo* Ventriculomegaly from True Hydrocephalus

One of the principal challenges in diagnosing iNPH is distinguishing it from ventricular enlargement secondary to cerebral atrophy or parenchymal loss, commonly referred to as *ex vacuo* hydrocephalus or ventriculomegaly. Because brain atrophy and impaired CSF dynamics may coexist in many patients, ventricular enlargement in older adults should not be conceptualized as a binary diagnostic entity. Rather, it represents a pathophysiological continuum spanning from states dominated by altered CSF dynamics and reduced brain compliance—typical of iNPH—through mixed neurodegenerative conditions, to ventricular expansion caused exclusively by loss of brain parenchyma (*ex vacuo* ventriculomegady).

Although intracranial volume remains relatively stable across the lifespan in both sexes, most studies indicate that men have larger brain volumes than women of similar age and health status, even after adjustment for body size [18]. Sex-related differences are also evident in the extent of brain tissue atrophy and CSF compartment expansion during normal aging [19]. The total CSF volume in adults averages approximately 335 mL, of which roughly 250 mL is contained within the intracranial compartment—comprising both the ventricular system and the cranial subarachnoid spaces—while the remaining ~85 mL fills the spinal subarachnoid space [20]. Intracranial CSF is unevenly distributed, with its total volume partitioned between the ventricular system (~33 mL) and the cranial subarachnoid spaces (~215 mL) [20]. However, age-related changes and substantial interindividual variability exist in the distribution of brain and CSF [18]. Conceptually, “possible” iNPH is only suspected in the presence of ventricular dilatation that is disproportionate to normal age-related changes. However, the interpretation of what constitutes “normal age-related changes” varies substantially among neurologists, neuroradiologists, and neurosurgeons. These divergent perspectives contribute to substantial variability in the identification of patients with suspected iNPH and, ultimately, in clinical decisions regarding shunt candidacy.

Defining what constitutes “normal” versus abnormal ventricular enlargement is further complicated by the substantial overlap between the clinical and neuroimaging features of possible iNPH and those of age-related cerebral atrophy, Alzheimer’s disease (AD), cerebrovascular white-matter disease, and parkinsonian neurodegenerative disorders [21]. Conventional neuroradiological markers of suspected iNPH—such as the EI, the callosal angle, and the DESH pattern [5]—are useful qualitative indicators but insufficiently sensitive or specific for the early identification of iNPH and for its distinction from other age-related causes of cognitive and gait impairment.

Ventricular dilatation has traditionally been assessed in clinical guidelines and the majority of published studies using the EI, a metric originally introduced by Evans in 1942 to quantify ventricular size on pneumoencephalography in pediatric populations [22]. The EI is calculated as the ratio of the maximal frontal horn width of the lateral ventricles to the maximal inner cranial diameter measured on the same axial slice [5]. Despite the discontinuation of pneumoencephalography and the well-recognized limitations of the EI in capturing three-dimensional ventricular geometry and total volume, the EI remains widely used in contemporary imaging modalities like computed tomography (CT) and magnetic resonance imaging (MRI). Its persistence is attributable to its simplicity and historical continuity, typically employing a cut-off value of 0.30 to indicate ventricular enlargement, as derived from Evans’ original description. Therefore, its primary role is as a screening tool, but it is insufficient for establishing a definitive diagnosis of possible iNPH.

An accurate differential diagnosis of ventricular enlargement is essential, because enlargement driven predominantly by parenchymal loss (true *ex vacuo* ventriculomegaly) is unlikely to respond to CSF diversion, whereas patients with true iNPH may experience meaningful clinical benefit from surgical treatment. Emerging evidence challenges a strict dichotomy, indicating that brain atrophy and impaired CSF dynamics coexist in some older patients, producing a mixed phenotype of hydrocephalus and neurodegenerative disease, most commonly AD. In a study we published that used ICP monitoring criteria to guide patient selection, we found that patients with iNPH who exhibited neuroradiological markers traditionally associated with poor prognosis could nevertheless experience meaningful postoperative improvement—particularly in gait and sphincter control [8]. Müller-Schmitz et al. also reported that patients with MRI findings compatible with iNPH who additionally exhibited CSF biomarker profiles suggestive of AD may still show significant improvement in cognitive symptoms and gait following a spinal tap test involving the removal of 40–50 mL of CSF [23]. Further supporting these findings, Ye et al. conducted a single-center retrospective study of 58 surgically responsive iNPH patients and reported that cortical brain biopsies taken during shunt placement, and immunostained for amyloid-β (Aβ) and phosphorylated tau, demonstrated Aβ-positive staining in 40% of cases [24]. Although Aβ-positive iNPH patients showed poorer cognitive outcomes at one year postoperatively, they nonetheless achieved significant functional improvement on the modified Rankin Scale [24].

To date, neither CT nor conventional MRI—nor even more advanced MRI techniques developed to assess glymphatic flow—have been able to reliably distinguish true *ex vacuo* ventriculomegaly from cases in which ventricular enlargement arises from a combination of impaired CSF dynamics and age-related changes in the elastic properties of the brain, as originally described by Salomón Hakim in 1965 [25,26]. This limitation is particularly relevant given that ventricular enlargement is more common in older adults than previously recognized. Using the EI as a screening measure for ventricular dilatation, a large, well-conducted population-based study of individuals aged ≥70 years in Sweden reported that 20.6% of participants had an EI greater than 0.30 on CT scans [27]. When neuroradiological criteria for iNPH were applied to the same population, 4.5% of individuals aged 70 years or older met guideline-based criteria for probable iNPH [28]. In an independent study employing an automated machine learning-based approach to detect the DESH neuroradiological phenotype—defined by ventriculomegaly with tight CSF spaces at the high convexity and enlarged Sylvian fissures—among ADNI-GO/2 participants, Günther et al. identified a DESH pattern in 7.9% of 270 cognitively normal individuals (mean age 72.9 years) [29]. According to the criteria of the third edition of the Japanese guidelines for iNPH, the presence of this phenotype suggests abnormal CSF dynamics and is consistent with a diagnosis of probable iNPH [5]. Whether individuals with an EI greater than 0.30 represent a preclinical phase of iNPH remains unknown and will require clarification through long-term longitudinal follow-up studies. In this complex scenario, assessing ventricular enlargement in older adults requires a new generation of precise and highly reproducible tools capable of improving diagnostic accuracy and identifying structural signatures that disentangle the respective contributions of normal aging, CSF abnormalities, and underlying neurodegenerative pathology. This is precisely where automated voxel-based morphometry (VBM) plays a critical role.

VBM enables tissue segmentation, the spatial normalization of individual MRI scans to a standardized anatomical template, and voxelwise statistical analysis [30]. It has been widely applied to the study of neurodegenerative disorders, including Alzheimer’s disease, mild cognitive impairment, frontotemporal dementia, dementia with Lewy bodies, and Parkinson’s disease [31]. A variety of automated and semi-automated software tools are available for voxel-based MRI volumetric analysis, including FreeSurfer, SPM-VBM, FSL, ANTs, and volBrain/vol2Brain [30,32,33,34,35,36]. Although these tools are open-source and/or freely available for non-commercial research use, they employ distinct methodological approaches, leading to differences in segmentation strategies, anatomical definitions, and output measures [30,32,33,34,35,36]. In addition, several commercially available proprietary solutions exist for both clinical and research applications. In a systematic review, Pemberton et al. identified 17 commercial volumetric products and noted that, despite widespread technical validation, comprehensive clinical validation and effective integration into real-world clinical practice remain limited [37].

Because providing a comprehensive review of available software is not the aim of this paper, we focused instead on a subset of reliable, freely accessible, non-commercial tools widely used and cited in voxel-based MRI volumetric analysis, specifically 3D Slicer, FreeSurfer, and volBrain. For readers interested in these tools, the paper by Harkey et al. provides an excellent and detailed review of all three [38]. However, manual or semi-automated VBM approaches are strongly influenced by image quality and registration accuracy, are labor-intensive, and depend heavily on operator expertise. Consequently, they introduce significant subjectivity and inter-operator variability and are generally practical only in specialized centers with dedicated expertise and infrastructure [39].

## 3. Principles and Clinical Relevance of AI-Based Brain Volumetry

Artificial intelligence (AI) began to be incorporated into VBM during the 2010s, in parallel with major advances in machine-learning (ML) and, most notably, the emergence of deep-learning (DL) methods. DL uses artificial neural networks with multiple hierarchical layers that are able to learn complex, non-linear representations from data by iteratively adjusting millions of parameters during training [40]. Over the past decade, artificial intelligence (AI) has also been incorporated into brain volumetry (hereafter referred to as AI-BrV), and this integration has substantially enhanced the accuracy and reproducibility of structural brain quantification. By automating key processing steps, AI-BrV has made sophisticated volumetric analyses accessible to clinicians and researchers with limited experience in manual segmentation or advanced image-processing software. As a result, AI-BrV has transformed brain volumetry from a labor-intensive, operator-dependent procedure into a highly automated and reproducible analytical tool. AI-BrV approaches fall into two main categories: traditional machine-learning (ML) methods and contemporary deep-learning (DL) techniques [41,42]. Early ML methods used atlas-based techniques that depended on manually selected features and expert-defined anatomical rules [43]. By contrast, DL approaches learn hierarchical anatomical features directly from large, expert-annotated datasets, resulting in marked improvements in segmentation accuracy, reproducibility, and generalizability across scanners and imaging protocols [44,45].

The main advantage of AI-BrV lies in its use of automated, algorithm-driven methods that apply advanced image-processing techniques to brain MRI scans, enabling unbiased, operator-independent, whole-brain structural assessments that overcome the regional limitations and subjectivity of manual or semi-automated approaches [32,46,47,48]. In the context of interpreting brain changes associated with aging and neurodegenerative diseases, these methods enable clinicians and researchers to identify characteristic patterns and imaging signatures of brain atrophy linked to normal aging, various neurodegenerative disorders, and iNPH.

## 4. The volBrain Platform and vol2Brain Pipeline

In the field of AI-BrV, the term software ‘pipeline’ refers to an automated and standardized sequence of computational steps that transforms raw MRI data into quantitative volumetric measurements [35]. By integrating pre-processing, segmentation, anatomical labeling, and volume estimation, these pipelines consistently generate highly reproducible results [35,47,48]. volBrain is an online, freely accessible neuroimaging platform (https://volbrain.net) that provides fully automated analysis of structural brain MRI data, with a particular emphasis on accurate and reproducible volumetric measurements [47,48]. By allowing users to upload MRI scans through a web-based interface, volBrain provides standardized outputs including brain tissue segmentation, subcortical and cortical volume estimates, and comparisons with age- and sex-adjusted normative reference populations. The platform operates without the need for local software installation and is intentionally designed to be user-friendly, thereby enabling clinicians and researchers to perform advanced brain morphometry with ease. volBrain has gained broad adoption in neuroscience and clinical research for investigating brain development, aging, and neurological diseases, and it supports transparency and methodological rigor in MRI-based volumetric analysis [47,48].

vol2Brain is the latest iteration of the volBrain processing pipeline. As a fully automated, cloud-based system, vol2Brain offers several practical advantages, including standardized processing, minimal user intervention, and easy accessibility without the need for local software installation [47,48]. In brief, vol2Brain enables rapid processing times on the order of minutes, in contrast to the many hours typically required by other VBM methods. This efficiency is achieved through a non-local, multi-atlas patch-based label fusion framework, integrated within a fully automated quality control and reporting pipeline [48].

**Figure 1:**
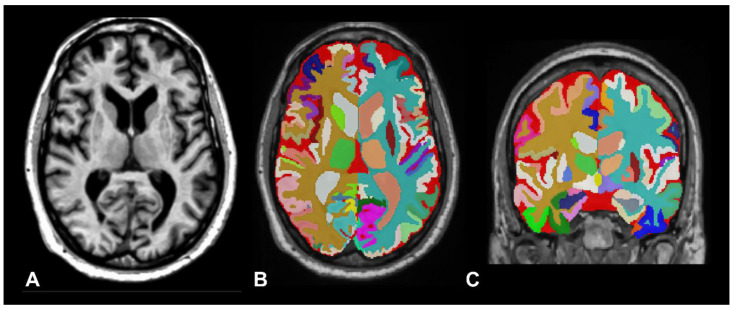
Healthy control subject from the Alzheimer’s Disease Neuroimaging Initiative (ADNI) database (ADNI reference: 168_S_6371). Data used in the preparation of this figure were obtained from the ADNI database (adni.loni.usc.edu). © ADNI. Used with permission. This 66-year-old male, with 14 years of education and a Mini-Mental State Examination score of 27, underwent MRI evaluation. The 3.0-T T1-weighted axial images were processed through the vol2Brain pipeline. (**A**) T1-weighted 3.0-T axial image. (**B**,**C**) Axial and coronal selected images of the structural segmentation generated by the vol2Brain report. In this patient, the total intracranial volume (T-ICV) was 1359 mL. The total brain volume (brain, cerebellum, and brainstem) was 1081.9 mL, corresponding to 79.6% of the T-ICV. Spaces in dark red correspond to extraventricular CSF, which in this healthy subject was 210.7 mL (15.5%).

**Figure 2:**
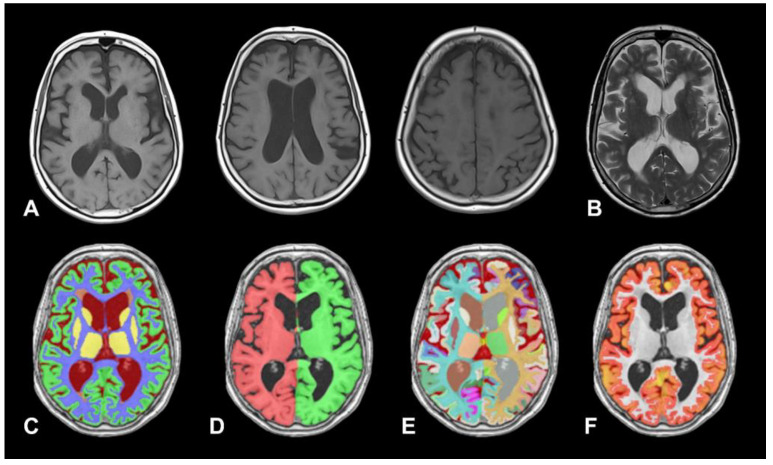
An 80-year-old woman presented with a one-year history of progressive gait instability and urinary incontinence, without subjective cognitive complaints. Brain MRI demonstrated ventriculomegaly with an Evans index of 0.34 and a reduced callosal angle of 70°, findings supportive of idiopathic normal pressure hydrocephalus (iNPH). To further evaluate the diagnosis and assess shunt responsiveness, she was admitted for continuous intracranial pressure monitoring. (**A**): T1 weighted MRI performed on a 1.5 T scanner demonstrates ventricular dilatation with obliteration of the paramedial convexity sulci. These findings are consistent with DESH criteria. (**B**): T2 weighted MRI images show leukoaraiosis involving the periventricular white matter. C-F: Axial images generated by the vol2Brain pipeline. (**C**): Tissue segmentation. The segmentation defines white matter (WM), cortical gray matter (GM, green), subcortical GM (yellow), cerebrospinal fluid (CSF, dark red), and the total intracranial volume. (**D**): Macrostructural labeling includes the cerebrum (right and left hemispheres), the cerebellum (including the vermis), and the brainstem (not shown). (**E**): Automatic structure segmentation. Delineation and labeling of 135 anatomical structures for detailed volumetric and structural assessment. (**F**): Cortical thickness report. Provide cortical thickness volume (in this case 314 mL), accompanied by a color coded visualization of thickness distribution, making it easier to interpret structural brain changes in which warmer colors (reds/yellows) typically represent thicker cortex and cooler colors (blues) represent thinner cortex.

To run the vol2Brain pipeline, users submit anonymized MRI scans in Neuroimaging Informatics Technology Initiative (NIfTI) format to the volBrain platform. The system first applies automated preprocessing procedures—such as denoising, intensity normalization, and global image-quality enhancement. The processed images are then spatially normalized to the Montreal Neurological Institute (MNI) template, ensuring robust anatomical alignment and facilitating consistent comparisons across different individuals, imaging protocols, and study cohorts [47]. Once preprocessing is completed, vol2Brain performs whole-brain parcellation, cortical thickness estimation, tissue segmentation—including white matter lesions—and intracranial cavity segmentation from T1-weighted MRI acquired at either 1.5 T or 3.0 T field strengths [47]. To illustrate these segmentation methods, Figure 1 presents results from a healthy control subject from the Alzheimer’s Disease Neuroimaging Initiative (ADNI), with selected quantitative measures summarized in Table 1. Figure 2 shows the analysis and corresponding output for a patient with iNPH, including a summary of the quantitative information generated by the tool.

When examining vol2Brain figures, it is important to note that the visual appearance of the segmentation outputs is intended primarily for quality control and validation rather than for detailed anatomical illustration. Similar to routinely used post-processing outputs in PET and SPECT neuroimaging, these images allow users to visually assess the plausibility of tissue classification and anatomical labeling. The absence of enhanced visual rendering does not reflect a limitation of the underlying methodology, which prioritizes accurate and reproducible quantitative measurements over visual presentation.

Following this step, vol2Brain performs intracranial cavity extraction, whole-brain segmentation, and automated anatomical labeling, generating a tissue-type segmentation map that includes multiple structures and tissue classes, such as the hippocampus, thalamus, cerebellum, cerebrospinal fluid, and cortical and subcortical gray matter. The pipeline also provides cortical thickness estimation [47]. Volumes of the segmented brain regions are reported both as absolute values and as relative measures expressed as a percentage of total intracranial volume (T-ICV) (Table 1). Results are returned to users by email or made available for direct download from the platform [47].

**Figure 3:**
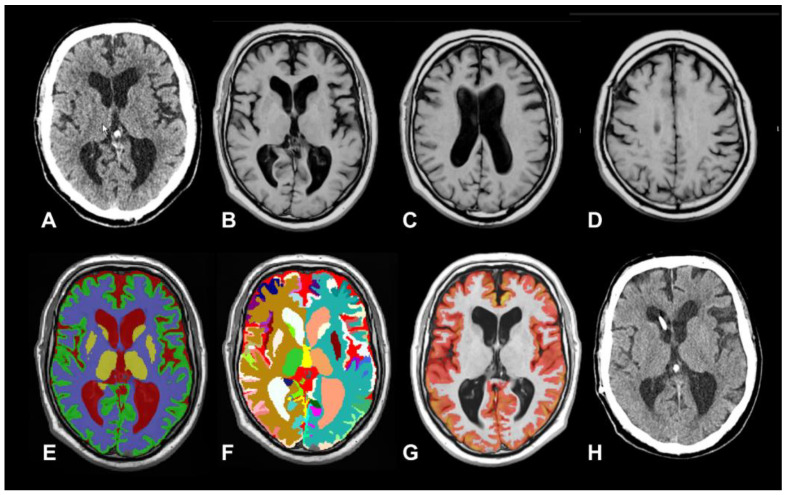
This 72-year-old man, with a medical history of controlled bipolar disorder, hypertension, type 2 diabetes, and a prior transient ischemic stroke, was referred to us by the neurologist after reporting that one year before referral he had begun experiencing progressive gait disturbances with frequent falls, cognitive impairment, and urinary incontinence. In the Addenbrooke’s Cognitive Examination III, the patient achieved a total score of 75, which falls below the commonly accepted dementia cut-off (≤82) and indicates significant cognitive impairment. (**A**): CT scan showing an Evans Index of 0.35; (**B**–**D**): Axial T1-weighted MRI at 1.5-T. The neuroradiological report described “moderate cerebral atrophy”, with small areas suggestive of moderate leukoaraiosis on T2-weighted images (not shown). (**E**): Axial vol2Brain output for brain tissue segmentation after registration of the uploaded T1 files to the Montreal Neurological Institute (MNI) template. CSF is shown in dark red, white matter in blue, cortical gray matter in green, and subcortical gray matter in yellow. (**F**): Structure segmentation. vol2Brain automatically identifies 135 brain structures, including lobes, ventricles, cerebellum, brainstem, and hippocampus, and assigns distinct color codes to each anatomical region; (**G**): Cortical thickness report and map generated with vol2Brain from T1-weighted MRI registered to the MNI template; regions of cortical thinning are highlighted in red. Total cortical gray matter volume was 528 mL. (**H**): CT scan one year after ventriculoperitoneal shunt placement, showing clinical improvement in gait stability and urinary continence.

**Figure 4:**
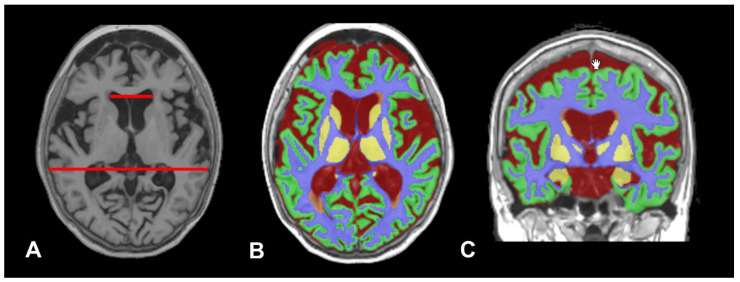
An Alzheimer’s disease patient case retrieved from the Alzheimer’s Disease Neuroimaging Initiative (ADNI) database (Patient ID: 003_S_1059). Data used in the preparation of this figure were obtained from the ADNI database (adni.loni.usc.edu). © ADNI. This 85-year-old woman had 18 years of education, a Mini-Mental State Examination score of 25, and a marked amnestic profile on Rey Auditory Verbal Learning Test. Her Clinical Dementia Rating-Sum of Boxes (CDR-SB) score was 6, consistent with mild dementia (CDR-SB range: 0–18). Lumbar CSF biomarkers were not consistent with Alzheimer’s disease pathology. Results: Aβ42 = 1306 pg/mL; total tau = 154.1 pg/mL; phosphorylated tau = 11.9 pg/mL. 1.5-T axial T1-weighted MRI images were processed through the vol2Brain pipeline. (**A**): Axial T1-weighted MRI acquired displayed an Evans Index of 0.27. (**B**): Axial tissue segmentation output generated by vol2Brain, illustrating automated classification of gray matter, white matter, and CSF. (**C**): Coronal tissue segmentation view. The processing yielded an estimated total intracranial volume (T-ICV) of 1377 mL. The patient’s total CSF volume was 391 mL, representing 28.4% of the T-ICV. Of this total, 74 mL were contained within the ventricular system, and 317 mL corresponded to the extraventricular CSF.

As already discussed, in iNPH, the central diagnostic challenge is distinguishing true hydrocephalus from ventriculomegaly secondary to age-related or neurodegenerative brain atrophy (*ex vacuo* ventriculomegaly). In neurodegenerative conditions, ventricular enlargement occurs passively as a consequence of progressive parenchymal volume loss. This *ex vacuo* expansion can closely mimic the radiological appearance of iNPH, despite the fundamentally different underlying mechanisms. In iNPH, ventricular dilation is driven primarily by impaired CSF absorption and/or altered CSF dynamics, together with changes in the poroelastic properties of brain tissue, rather than by tissue loss. These overlapping imaging features often complicate diagnosis and highlight the need for adjunctive physiological assessments and multimodal evaluation. In this context, AI-BrV provides a valuable tool for distinguishing between these conditions by enabling anatomically precise quantification of ventricular enlargement and associated parenchymal changes. Beyond absolute ventricular volume measurements, AI-BrV–derived data can be used to construct composite indices and ratios—such as ventricular-to-intracranial volume ratios, metrics of periventricular white matter integrity, cortical and subcortical gray matter profiles, and deep gray matter atrophy patterns—which may help to define distinct structural phenotypes in brains with ventricular dilatation (Figure 3 and Figure 4).

Segura-Hernández et al., using vol2Brain, conducted pre- and post-shunt volumetric analyses in 30 patients with iNPH who underwent ventriculoatrial shunt surgery, of whom 26 (86.7%) showed improvement in gait disturbance following the procedure [49]. Volumetric analysis revealed a statistically significant increase in white matter, gray matter, and total brain parenchymal volumes (mean increase of 4.2%), accompanied by a significant reduction in total cerebrospinal fluid volume at a mean follow-up of 9.3 months post-shunt [49]. The authors introduced the term “cerebral pseudoatrophy” to describe this unexpected postoperative increase in brain parenchymal volume [49]. In this setting, AI-BrV may play a central role in distinguishing cerebral pseudoatrophy from true neurodegenerative atrophy. By providing anatomically precise measurements of ventricular size, global and regional brain parenchymal volumes, and tissue-specific changes over time, volumetric analyses allow differentiation between passive ventricular enlargement due to irreversible parenchymal loss and potentially reversible compression related to altered CSF dynamics. Longitudinal volumetric assessment before and after shunt surgery is particularly informative: increases in brain parenchymal volume accompanied by corresponding reductions in CSF volume following CSF diversion are inconsistent with true atrophy and instead support pseudoatrophy. These objective, quantitative metrics offer a robust framework for addressing this key diagnostic question in iNPH.

## 5. Beyond Ventricles and Cortex

In AD, global brain atrophy is accompanied by ventriculomegaly and the involvement of structures such as the entorhinal cortex, hippocampus, caudate, and temporal lobe. Atrophy in these regions correlates closely with cognitive decline and serves as an imaging biomarker of disease progression [50]. In iNPH studies, structural assessment has focused predominantly on ventricular enlargement and the degree of cortical atrophy. In contrast, subcortical structures—particularly the basal ganglia and other deep gray matter nuclei—have received comparatively little attention, despite their central role in gait control, cognition, and motor integration.

A few studies have suggested that deep gray matter structures are affected in patients with iNPH. Ishii et al. identified decreased gray matter density in the insula, caudate nucleus, and thalamus in 34 patients with iNPH compared with age-matched healthy controls and an equally sized group of patients with AD [51]. Virhammar et al. using quantitative arterial spin-labeling MRI in patients with iNPH, reported significant bilateral reductions in perfusion within the lentiform nucleus, thalamus, and periventricular white matter compared with healthy age-matched controls [52]. Deep gray matter abnormalities involve key nodes of the basal ganglia–thalamocortical circuits and may represent a structural substrate underlying the gait disturbances, psychomotor slowing, and executive dysfunction characteristic of iNPH. We hypothesize that selective involvement of these circuits contributes to the potentially reversible clinical phenotype of iNPH and distinguishes it from neurodegenerative atrophy. In this context, AI-BrV provides a powerful framework for systematically comparing patients with iNPH, AD, and healthy older adults by enabling detailed, objective characterization of the basal ganglia and other deep gray matter structures. Such analyses may help to identify disease-specific morphological patterns, elucidate mechanisms underlying symptom reversibility after CSF diversion, and refine imaging-based phenotypes across disorders that share overlapping clinical features. By quantifying subtle regional differences and tracking structural trajectories over time, AI-BrV will contribute to a more granular understanding of both neurodegenerative and hydrocephalic processes, thereby supporting a more accurate and reliable differential diagnosis.

The importance of CSF biomarkers in neurodegenerative diseases has increased substantially over the past decade, driven largely by the widespread adoption of standardized panels specific to AD. These panels primarily target two hallmark proteins of AD: β-amyloid and phosphorylated tau. Measurement of β-amyloid 1–42 is particularly informative, as reduced CSF levels reflect its pathological sequestration into amyloid plaques within the brain. This biomarker serves as a core diagnostic indicator of AD and shows strong concordance with amyloid PET imaging, which directly quantifies cortical β-amyloid deposition. The resulting biochemical signature supports more accurate and earlier diagnosis, often preceding detectable cognitive decline or structural brain changes [53]. However, a notable diagnostic discordance has been reported in AD. Studies indicate that in approximately 20% of cases, there is a mismatch between CSF Aβ42 levels and amyloid-PET findings [54]. This discrepancy occurs not only in patients with AD but also among cognitively normal controls [54,55].

The relevance of CSF β-amyloid levels in patients with iNPH is already under scrutiny. This interest is driven by the frequent coexistence of AD in older adults with iNPH and by the observation that CSF Aβ42 levels may be reduced in iNPH patients even in the absence of true amyloid plaque deposition. Several studies have shown that impaired CSF dynamics and ventricular enlargement in iNPH can lead to altered distribution, clearance, or dilution of amyloid peptides, potentially mimicking the biochemical profile typically associated with AD. This concept is supported by evidence demonstrating that restoration of CSF flow following shunt surgery modifies amyloid-β behavior and shifts Aβ species in the CSF, indicating that CSF flow abnormalities in iNPH significantly influence amyloid transport and clearance [56]. In a study conducted within the Mayo Clinic Study of Aging, Graff-Radford et al. analyzed individuals with a CSF+/PET− biomarker profile [55]. This profile refers to cases in which β-amyloid levels are abnormally low in the CSF while amyloid-PET imaging does not show increased β-amyloid deposition. Their findings demonstrated that this discordant biomarker pattern is associated with features of impaired CSF dynamics—specifically, imaging markers of DESH—suggesting that alterations in CSF circulation can confound the interpretation of amyloid biomarkers [55]. In this study, a higher DESH score correlated with a greater likelihood of CSF+/PET− status, suggesting that an increased total CSF volume may induce a dilutional effect that confounds the true value of amyloid biomarkers [55].

The use of AI-BRV could help to refine biomarker thresholds by adjusting them to an individual’s total CSF volume. This approach would partially account for the dilutional effects associated with abnormal CSF dynamics in patients with iNPH, as well as in individuals with *ex vacuo* ventriculomegaly, in whom total CSF volume is also increased. In this context, extracerebral CSF volumes should also be considered in order to develop biomarker thresholds that are physiologically meaningful and robust to interindividual variation in CSF compartment size. Relying on uncorrected CSF β-amyloid levels likely contributes to an overestimation of amyloid pathology when interpreting CSF and PET studies in individuals with ventriculomegaly.

## 6. The Road Ahead: Toward an Integrated Framework in iNPH

As argued by Sir Muir Gray, the principal barrier to improving healthcare outcomes lies not in the generation of new knowledge but in the failure to incorporate what we already know into routine clinical practice [57]. Clinical research in iNPH has expanded markedly over the past two decades, particularly in CSF biomarkers, ICP monitoring, CSF dynamics, and guideline development. Yet, despite this progress, interpretation of findings remains inconsistent across professional groups. This lack of consensus contributes to unwarranted variation in clinical practice and delays in the adoption of effective screening tests, diagnostic pathways, and therapeutic interventions. This discrepancy is further reinforced by a long-standing paradox frequently raised by so-called *iNPH skeptics*: definitive confirmation of the diagnosis—“confirmed iNPH”—is often established only retrospectively, based on a patient’s postoperative clinical response, rather than on independently validated preoperative diagnostic criteria [7,11,12,16]. In this context, AI-BrV may offer objective and reproducible metrics that help to bridge the gap between preoperative uncertainty and postoperative confirmation. By providing more reliable indicators of disease status, it has the potential to reconcile divergent viewpoints and contribute to a more standardized and evidence-based framework for patient selection.

A well-recognized paradox is that many patients with iNPH who experience favorable clinical outcomes show no corresponding reduction in ventricular size on traditional two-dimensional measures such as the EI [58,59]. Meier and Mütze, in a cohort of 80 shunted patients with iNPH, reported that the majority (80%) showed no detectable change in the EI one year after surgery [59]. Even so, 59% of these patients achieved excellent clinical outcomes, and an additional 17% demonstrated satisfactory improvement, indicating that clinical benefit did not correlate with postoperative reductions in ventricular size [59]. This finding aligns with the broader understanding that the EI—like most two-dimensional indices—provides only a crude approximation of ventricular size and does not account for the extraventricular CSF compartment; consequently, it is a poor surrogate for total intracranial CSF volume [58]. By contrast, AI-BrV captures three-dimensional changes in ventricular CSF and quantifies both intraventricular and extraventricular CSF compartments, enabling a more sensitive assessment of CSF redistribution that may correlate more closely with clinical improvement following shunt surgery [58]. In addition, AI-BrV enables the automatic derivation of novel brain–CSF metrics and ventricular-to-extraventricular CSF ratios (Table 1), as reported in pilot studies using quantitative MRI [60]. Consequently, establishing whether intracranial CSF volume is significantly reduced in shunted patients with iNPH, and assessing the extent to which these changes correlate with clinical outcomes, requires further investigation using these advanced tools.

Autopsy series, cortical biopsy studies obtained during shunt placement, and CSF biomarker analyses consistently indicate that approximately one-third to one-half of patients with iNPH exhibit amyloid-β deposition, tau pathology, or both. However, the presence of amyloid-β pathology does not necessarily rule out a favorable functional outcome after shunt surgery [24,61]. This overlap suggests that ventricular enlargement in older adults often results from a combination of impaired CSF dynamics and underlying neurodegenerative processes. A major strength of AI-BrV pipelines lies in their capacity to reanalyze large existing databases and the extensive neuroimaging datasets accumulated over the past decade in well-characterized patient cohorts, such as those from the ADNI. In addition, many centers have established registries of iNPH patients treated with CSF shunting, most of whom underwent high-quality imaging—typically with 1.5-T MRI scanners—and were evaluated using heterogeneous neuroimaging criteria and diagnostic frameworks. These legacy datasets—encompassing both shunt responders and non-responders—offer a unique opportunity for systematic reanalysis using AI-based BrV pipelines. Leveraging such data may facilitate the development of more robust predictive models and, ultimately, improve patient selection and therapeutic decision-making in iNPH. Quantitative features extracted from AI-BrV output—such as ventricular and extraventricular CSF volumes, cortical and subcortical gray-matter measures, and periventricular white-matter metrics—can be integrated into multivariable statistical and machine-learning frameworks, including logistic regression, linear mixed-effects models, and tree-based classifiers. These approaches may help to identify neuroimaging biomarkers capable of distinguishing shunt responders from non-responders. Overall, AI-BrV should be viewed as an initial step toward developing robust and generalizable decision-support tools for iNPH. Its potential for external validation across centers, combined with iterative refinement through larger multicenter studies, positions it as a promising tool for future clinical integration.

AI-BrV enables the automated labeling of cortical layers, deep gray matter nuclei, periventricular white matter, and microstructural alterations with unprecedented reliability and anatomical detail. When applied to large cohorts of patients with iNPH, AI-BrV supports normative modeling by enabling the derivation of normative deviation scores (e.g., z-scores), allowing quantitative comparisons with internal control groups and large publicly available neuroimaging repositories such as ADNI, OASIS, and the UK Biobank [62,63].

## 7. Conclusions

iNPH remains a diagnostically challenging and frequently underrecognized condition, in part because ventricular enlargement in older adults reflects a continuum of overlapping pathophysiological processes rather than a discrete binary entity. AI-based brain volumetry offers an objective framework for quantifying three-dimensional structural alterations across ventricular, extraventricular CSF, and brain parenchymal compartments, addressing key limitations of conventional neuroradiological markers. By enabling data-driven disease phenotyping, retrospective harmonization of legacy datasets, and external validation against large normative cohorts, AI-BrV may help to reconcile divergent clinical interpretations and refine patient stratification.

As volumetric pipelines become increasingly standardized and integrated with clinical measures, physiological assessments, and panels of CSF biomarkers, such approaches may contribute to re-establishing iNPH as a biologically grounded and therapeutically relevant disorder with the potential for the reversibility of cognitive and gait impairment in aging populations.

## Figures and Tables

**Table 1:** Volumetric data extracted from the vol2Brain report of a 66-year-old male participant from the Alzheimer’s Disease Neuroimaging Initiative (ADNI reference: 168_S_6371; the images are shown in Figure 1). Volumes are reported in millilitres (mL) and, in the final column, as percentages of total intracranial volume (T-ICV). The ventricular-to–total CSF volume index (V-tCSF-I) was calculated as the ratio of intraventricular CSF volume to total intracranial CSF volume, including both intraventricular and extraventricular compartments. The brain parenchymal fraction (BPF%) represents the proportion of intracranial volume occupied by brain tissue and was calculated as (brain volume/T-ICV) × 100) and is expressed as a percentage.

vol2Brain|Structures and Tissues	Volume (mL)	Percent (%)
**Total gray matter (GM)**	**603**	**44.4**
Cortical GM	461.9	33.9
Subcortical GM	38.3	2.8
**Total white matter (WM)**	**478.9**	**35.3**
**Total intracranial CSF**	**259.5**	**19.1**
Intraventricular CSF	48.8	3.6
Extraventricular CSF	210.7	15.5
**Caudate**	6.7	0.49
**Putamen**	7.3	0.54
**Pallidum**	2.6	0.19
**BrainVol** **|** **BPF%**	1081.9	79.6
**V-tCSF-I**	-----	18.8
**Total intracranial volume (T-ICV)**	**1359**	**100%**

## Data Availability

Data used in preparation of this article (Figure 1 and Figure 4) were obtained from the Alzheimer’s Disease Neuroimaging Initiative (ADNI) database (adni.loni.usc.edu). As such, the investigators within the ADNI contributed to the design and implementation of ADNI and/or provided data but did not participate in analysis or writing of this report. A complete listing of ADNI investigators can be found at http://adni.loni.usc.edu/wp-content/uploads/how_to_apply/ADNI_Acknowledgement_List.pdf.

## Abbreviations

The following abbreviations are used in this manuscript:

Aβ	Amyloid-β
AD	Alzheimer’s disease
ADNI	Alzheimer’s Disease Neuroimaging Initiative
AI	Artificial intelligence
AI-BrV	AI-based brain volumetry
BPF	Brain parenchymal fraction
CSF	Cerebrospinal fluid
CT	Computed tomography
DESH	Disproportionately enlarged subarachnoid space hydrocephalus
DL	Deep learning
EI	Evans Index
GBD	Global Burden of Disease
GM	Gray matter
ICP	Intracranial pressure
iNPH	Idiopathic Normal Pressure Hydrocephalus
ML	Machine learning
MNI	Montreal Neurological Institute
MRI	Magnetic resonance imaging
NIfTI	Neuroimaging Informatics Technology Initiative
OASIS	Open Access Series of Imaging Studies
SPM	Statistical Parametric Mapping
T-ICV	Total intracranial volume
VBM	Voxel-Based Morphometry
VHIR	Vall d’Hebron Institut de Recerca
VHUH	Vall d’Hebron University Hospital
V-tCSF-I	Ventricular-to-total cerebrospinal fluid volume index
WM	White matter
